# On-Chip Organoid Formation to Study CXCR4/CXCL-12 Chemokine Microenvironment Responses for Renal Cancer Drug Testing

**DOI:** 10.3390/bios12121177

**Published:** 2022-12-17

**Authors:** Adem Ozcelik, Burcin Irem Abas, Omer Erdogan, Evrim Cevik, Ozge Cevik

**Affiliations:** 1 Department of Mechanical Engineering, Aydın Adnan Menderes University, Aydin 09010, Turkey; 2 Department of Biochemistry, School of Medicine, Aydin Adnan Menderes University, Aydin 09010, Turkey; 3 Department of Machinery and Metal Technologies, Kocarli Vocational School, Aydin Adnan Menderes University, Aydin 09010, Turkey

**Keywords:** organoid, microfluidics, droplet generation, renal cancer, tumor organoid

## Abstract

Organoid models have gained importance in recent years in determining the toxic effects of drugs in cancer studies. Organoid designs with the same standardized size and cellular structures are desired for drug tests. The field of microfluidics offers numerous advantages to enable well-controlled and contamination-free biomedical research. In this study, simple and low-cost microfluidic devices were designed and fabricated to develop an organoid model for drug testing for renal cancers. Caki human renal cancer cells and mesenchymal stem cells isolated from human umbilical cord were placed into alginate hydrogels. The microfluidic system was implemented to form size-controllable organoids within alginate hydrogels. Alginate capsules of uniform sizes formed in the microfluidic system were kept in cell culture for 21 days, and their organoid development was studied with calcein staining. Cisplatin was used as a standard chemotherapeutic, and organoid sphere structures were examined as a function of time with an MTT assay. HIF-1α, CXCR4 and CXCL-12 chemokine protein, and CXCR4 and CXCL-12 gene levels were tested in organoids and cisplatin responses. In conclusion, it was found that the standard renal cancer organoids made on a lab-on-a-chip system can be used to measure drug effects and tumor microenvironment responses.

## 1. Introduction

Organoid models are advanced methods that contribute to the development of tissue engineering to understand the mechanisms and events that occur in tissues. In recent years, organoid models have made significant strides in developing stem cell technology [1] and understanding cancer cellular development. Modeling studies of tissue organization in organoid models first started by mimicking intestinal fragments in colon tissue [2]. They then gained momentum in tumor modeling studies [3]. Organoids are needed to replicate patient-derived organoids and elucidate mechanisms and personalized treatment studies [4].

Different culture conditions for organoid production from tumors [5] and renal tumor biobanks have begun to be established in order to eliminate differences in tissue morphology [6]. Renal cancers are seen in adults and constitute about 3% of cancers and 70–80% of these are seen as clear cell carcinoma in renal cancer [7,8]. The essential feature of clear cell renal cells carcinoma is the high expression of HIF-1α, which includes both epithelial and mesenchymal characteristics [9]. Therefore, they are used in tumoroids formation and models from xenotransplantation as they continue to increase [10]. Because of the tendency to metastasize in different subtypes of renal cancer, radical surgery in one kidney is among the first treatments that come to mind [11]. However, the prognosis is poor because renal cancer’s survival rate is low compared to its stages. In studies conducted to date, organoid models for kidney cancer have not been successful enough and the methodology still needs to be expanded [12]. Renal organoid model studies started with the demonstration that embryonic stem cells can transform into renal cells when stimulated by nephrogenic factors [13]. Later, it was reported that they could form metanephric mesenchyme and ureteric bud by providing a 3D tubular structure using human pluripotent stem cells (hPSCs) [14]. Factors such as the ability to work in patient-derived organoid models depending on patient tissue, DNA sequencing differences, and intra- and inter-tumoral heterogeneity, create limitations in the 3D model [15,16]. The development of organoids has gained momentum due to the lack of reliable preclinical models for testing the sensitivity and dose of drugs in kidney cancer studies [17]. However, the patient-derived organoid model is still inadequate for extensive drug screenings. In particular, there is a need for the organoid models to be the same in every passage, to be stable in size, and to be able to compare the tested drugs and drug interactions as previously exhibited in primary tumors [12].

Miniaturization of conventional laboratory equipment and processes created a paradigm shift in sample manipulation and analysis [18,19]. For example, the field of microfluidics enabled numerous tools and new approaches for generation and study of organoids [20]. Microfluidic devices make essential contributions to evaluating organoids’ stability and environmental conditions. For example, organoid modeling studies have been carried out with 3D-printed fluidic chips about the kidney being a filtration organ and maintaining its viability in a flow due to vascularization in nephrogenesis [21]. The major benefits of the microfluidic methods are high precision, minimized contamination, and ease-of-operation that can be critical for biomedical research [22,23,24,25,26]. For example, Zheng et al. developed an acoustofluidic platform to study human brain organoids by implementing acoustic tweezers in microfluidic devices [27]. The miniaturized approach used in their work enabled the study of mini-bioreactors to form organoids to study human brain functions [28]. High-precision capabilities of microfluidic tools are also applied in precise manipulation of organoids to achieve complex tissue formations with controlled spatial manipulation. For example, human forebrain organoids and human midbrain organoids are shown to be fused using precise acoustofluidic manipulation to study the development of the human midbrain-to-forebrain mesocortical pathway [29]. Besides their precision, droplet microfluidic platforms are also implemented for high throughput generation of mammary tumor organoids by forming cell complexes within alginate microbeads [30]. Even though microfluidic platforms offer various benefits including precision, high throughput, and simplicity in organoid research, their complex and costly fabrication requirements limit their wide-spread implementation in low-resourced institutions. Therefore, more research should be done to demonstrate the feasibility of low-cost microfluidic device fabrication for organoid research applications. 

This study aims to determine the potential of using renal cancer cells and mesenchymal stem cells to be used in drug screening by developing an organoid model with uniform sizes that can mimic the tumor microenvironment by employing simple and low-cost microfluidic devices. For this task, a droplet microfluidic device is fabricated by a rapid laser prototyping approach. Low-cost laser prototyping and device preparation using poly (methyl methacrylate) (PMMA) is shown to be a reliable approach for fabricating microfluidic devices to form organoids and study drug testing for renal cancer tumors. 

## 2. Materials and Methods

### 2.1. Microfluidic Device Fabrication

A microfluidic device was designed in a computer-aided design software as a three-layer structure. The top, middle, and bottom layers show the inlet and outlet holes, the outline of the fluidic channel, and the lower boundary of the microfluidic device in two dimensions, respectively. The optically clear PMMA sheets (Polinyapi, Istanbul, Turkey) were chosen for this study. The height of each layer was determined by the height of the PMMA sheets. The heights of the middle fluidic layer, top layer, and bottom layer were set to be 500 µm, 2 mm, and 500 µm, respectively. The top layer was chosen to be 2 mm for more stable tubing connections. The bottom layer was chosen to be as thin as possible for better microcopy imaging. After each layer was cut in a 100-Watt CO_2_ CNC laser cutter (Lazerfix, 100 W, Konya, Turkey), chloroform (FKM Fine Plexy Adhesive, Lazerci, Istanbul, Turkey) vapor was applied between each layer for 10 min at room temperature. After chloroform treatment, the PMMA layers that were softened by surface solvation were pressed together using a manual presser at an estimated pressure of 50 psi for 10 min at approximately 60 °C. The prepared devices were baked at 50 °C overnight to provide a permanent and liquid-tight bonding. Polyethylene tubes with 1 mm inner diameter and 1.8 mm outer diameter (Hongzhe Industry Co., Shanghai, China) were inserted into the inlets and the outlets of the device and glued using a fast curing two-component epoxy for improved stability. After device assembly, the fluidic channel was flushed with copious amount of PBS solution. 

### 2.2. Cell Culture Conditions

In the study, Caki cells are human renal cancer cells known as epithelial morphology and clear cell carcinoma and were commercially obtained from ATCC (HTB-46). Caki cells were grown using McCoy’s 5a Medium Modified (Sigma Aldrich, Darmstadt, Germany) medium containing 10% FBS (26140079, Gibco, Waltham, MA, USA), Penicillin-Streptomycin (Sigma) (100 units). Human-derived mesenchymal stem cell (MSC), which we isolated from human umbilical cord tissue in our previous study, characterized and stored in the 3rd passage, was used as the source of mesenchymal stem cells [31]. MSC cells were grown DMEM/F12 (Gibco, Waltham, MA, USA) supplemented with 15% FBS, Penicillin-Streptomycin (100 units), L-Glutamine, and NaHCO_3_. It was allowed to incubate at 37 °C, in an environment containing 95% humidity and 5% CO_2_. 

### 2.3. Organoid Culture with Alginate Beads

Sodium alginate powder (W201502, Sigma Aldrich, Darmstadt, Germany) was dissolved in Ca-free and Mg-free DPBS buffer (14190144, Gibco, Waltham, MA, USA), and its final concentration was adjusted to 0.8% *w*/*v* [32]. Caki cells (2 × 10^4^) and MSC cells (2 × 10^4^) were mixed in PBS and infused from the middle inlet of the microfluidic device. Alginate, 0.1 M CaCl_2_ solution, mineral oil (M8410, Sigma Aldrich, Darmstadt, Germany) with 5 wt% SPAN 80, and cell solution were infused from the five inlets of the microfluidic device at 20 µL/min, 20 µL/min, 50 µL/min, 50 µL/min, and 10 µL/min, respectively, using a multi-channel home-built syringe infuser [33] and a commercial syringe pump (NE-1600, New Era, New York, NY, USA). The cell-laden alginate hydrogel beads were kept in sterile conditions in a 24-well plate and washed with sterile PBS. Alginate beads containing Caki and MSC cells were grown in McCoy and DMEM/F-12 mixed medium (ratio 2:1) at 37 °C and 5% CO_2_ conditions. Cells were followed for 21 days and replaced with fresh medium every 4 days. In addition, some of the alginate-capsulated Caki and MSC cells were grown in a cell medium containing 2 µM cisplatin on the 1st, 7th, 14th, and 21st days. As a cell control group, Caki cells were used with and without cisplatin, and the experiment results were compared to these cells.

### 2.4. Cell Viability with MTT Assay

MTT test was performed to determine the Cisplatin IC50 value at 24 h, 48 h, and 72 h on Caki cells. Cells were seeded in 1 × 10^4^ numbers in a volume of 200 µL on 96-well plates. Cisplatin was incubated at concentrations of 0.1–1000 µM for 24 h, 48 h, and 72 h. After incubation, the cell medium was discarded, and a fresh DMEM medium was added. Next, 5 mM MTT (3-(4,5-dimethylthiazol-2-yl)-2,5-diphenyltetrazolium bromide) solution (ODC Research and Development Inc., Aydin, Turkey) was added to the cells and incubated for 4 h. After incubation, 10 µL of DMSO was added, and the formazan crystals were dissolved. Absorbance values were read at 540 nm in an ELISA cover. The experiments were run in triplicate, and the IC50 value was calculated for Cisplatin.

### 2.5. Calcein Dye Staining

The viability of renal organoid cells in alginate beads was measured by Calcein staining [34]. Alginate-encapsulated cells with or without cisplatin (2 µM) were stained with calcein (C0875, Sigma Aldrich, Germany) on days 1, 7, 14, and 21. At the end of the incubation, the cells were incubated at 37 °C for 20 min with buffer containing (94 mM NaCI, 350 mM Na-Citrate, 35 mM MOPS) for solubilization of alginate. Then, calcein dye was added at 2 µg/mL concentration and incubated for 15 min at room temperature. After incubation, the dye was discarded by centrifugation, and the pellet was washed with PBS. Then, the pellet was suspended with PBS and read on the fluorescent reader (ex/em: 495 nm/635 nm) on a 96-well plate. Calcein is not a fluorescent dye, but due to its high esterase activity in living cells, it turns green fluorescent by esterases. 

### 2.6. HIF-1α, CXCR4 and CXCL-12 Protein Levels Measurement

First, the cells were removed from the alginate pellet by solubilization with buffer (94 mM NaCI, 350 mM Na-Citrate, 35 mM MOPS) to measure CXCR4 and HIF-1α protein levels. Cell pellets were lysed with cell lysis buffer containing NP40 and Triton X-100. In addition, CXCR12 protein levels were measured in the cell medium of the measurements. CXCR4, CXCL-12, and HIF-1α levels were determined using the ELISA kit according to the manufacturer’s protocol. Human CXCR4 sandwich ELISA kit (EH2136, sensitivity 46.875 pg/mL), Human HIF-1α sandwich ELISA kit (EH0551, sensitivity 94.00 pg/mL), and Human CXCL-12 sandwich ELISA kit (EH3755, sensitivity 94.00 pg/mL) were used (Fine Biotech Co., Wuhan, China), and the manufacturer’s protocol was followed. 

### 2.7. CXCR4 and CXCL-12 Gene Expression Measurement

For CXCR4 and CXCL-12 gene expression, cell pellets were kept in RNA lysis buffer in a sonicator for 30 s on ice using our previous study [31]. Then, total RNA was isolated and purified according to the kit protocol. Then, RNAs were translated into cDNA with the help of a reverse transcriptase kit. cDNA concentration was measured in nanodrop, Sybr Green master mix was used with 100 ng of cDNA, and qPCR was set up. Sequences of CXCR4 Forward 5′-TGACGGACAAGTACAGGCTGC -3′, CXCR4 Reverse 5′-CCAGAAGGGAAGCGTGATGA-3′; CXCL12 Forward 5′-TGCCAGAGCCAACGTCAAG-3′, CXCL12 Reverse 5′-CAGCCGGGCTACAATCTGAA-3′; GAPDH Forward 5′-AGGGCTGCTTTTAACTCTGGT-3′, Reverse 5′-CCCCACTTGATTTTGGAGGGA-3 primers were used for the reaction. Programmed was used at 95 °C for 5 min, then for 40 cycles at 95 °C for 15 s, 59 °C for 1 min, and 72 °C for 30 s using Sybr Green PCR Master Mix (Applied Biosystems, Foster City, CA, USA) in the ABI StepOne Plus detection system. Analyses were performed according to the 2^−ΔΔCt^ method using software, and GAPDH was used as a housekeeping gene.

### 2.8. Statistical Analysis

All experiments were carried out with at least three replications. Statistical analyses were performed using GraphPad Prism 7.0. Comparisons between groups in the analyses were made using the independent *t*-test, and the significance levels were accepted as values below 0.05.

## 3. Results

### 3.1. Microfluidic Device and Working Principle

**Figure 1:**
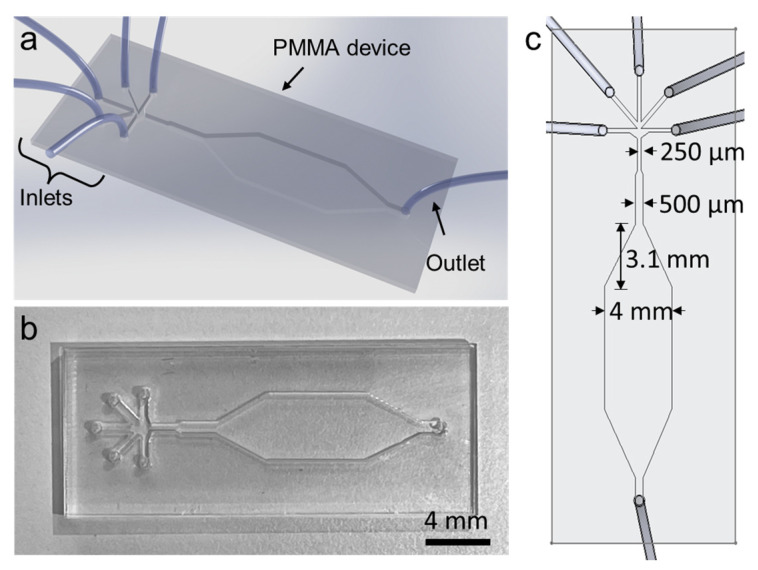
Fabricated PMMA device. (**a**) Schematic of the microfluidic device. (**b**) Actual picture of the microfluidic device fabricated using PMMA. (**c**) Detail dimensions of the microfluidic device (drawn to scale).

**Figure 2:**
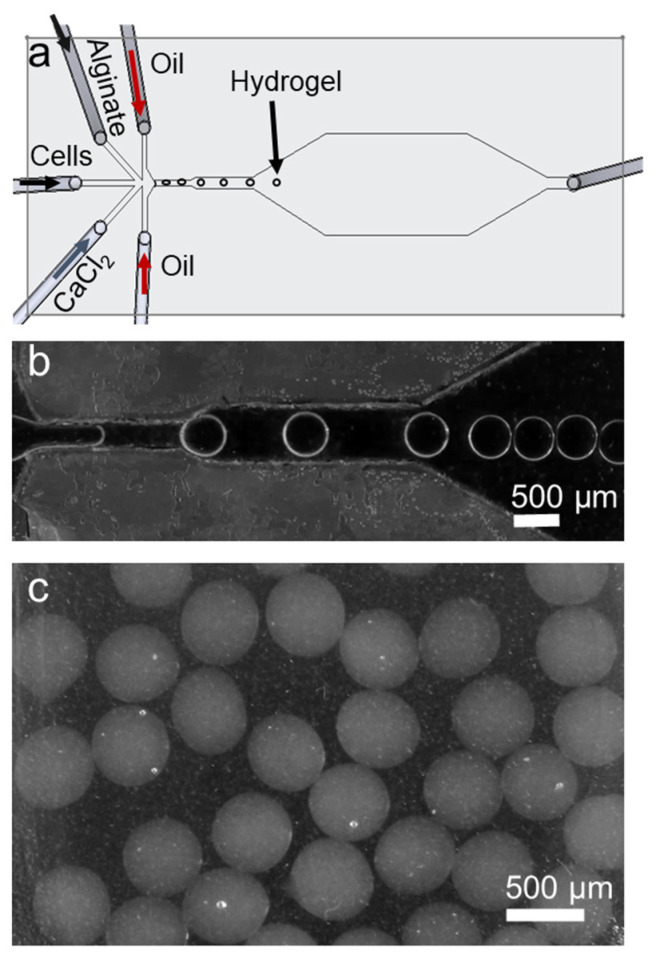
Working principle of the microfluidic device. (**a**) Device outline and injected fluids are shown. (**b**) Generated alginate hydrogel droplets are shown. (**c**) Collected alginate beads are shown.

Schematics and actual images of the fabricated microfluidic device are shown in Figure 1. The PMMA device is optically clear at the fluidic channel region and slightly opaque around the regions that were exposed to the chloroform vapor unevenly. The microfluidic device was completely sealed after bonding, preventing any liquid leakage. Five inlets of the device were designed to allow infusing of multiple fluids including cell solution, alginate, calcium chloride, and mineral oil. The working principle of the device is shown in Figure 2. The microfluidic device is designed to generate water-in-oil droplets. In this device, cell-laden alginate bead formation mechanism relies on alginate polymer crosslinking with calcium ions. This crosslinking occurs inside a droplet of alginate, cells, and calcium chloride mixture in an oil carrier phase. After the alginate beads are formed, they are washed with PBS and transferred into cell culture medium in well-plates. 

### 3.2. Characterization of Organoids

**Figure 3:**
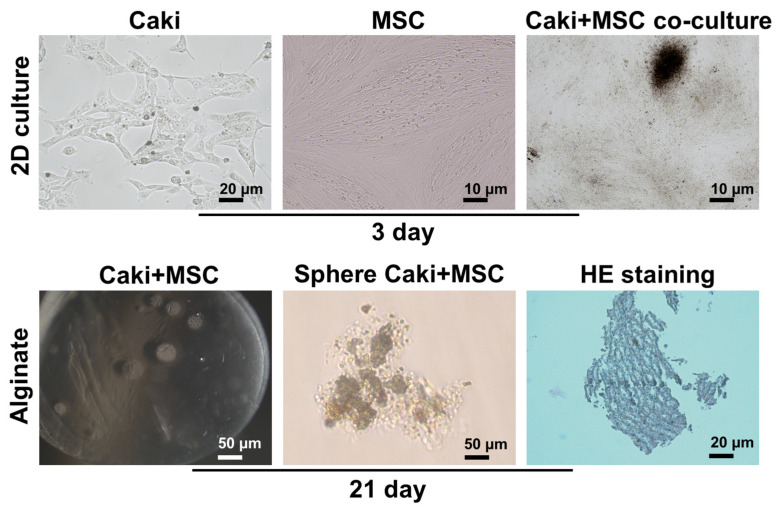
Characterization of organoid modelling developed from Caki and MSC cells and treatment by Cisplatin. Microscopic images showing growth of Caki and MSC cells and Caki-MSC co-culture cells on 3rd day (top row). Alginate beads organoid showing sphere formation, hematoxylin and eosin (H&E) staining on 21st day (bottom row).

Kidney-derived human clear cell carcinoma cells, Caki and MSC, isolated from human umbilical tissue were used to construct a renal cancer organoid model. The average size of the alginate hydrogel beads was measured to be 485 ± 45 µm (error represents the standard deviation of 30 beads). The morphological images and structures of the cells were checked under the microscope on the 3rd day by making a 2D culture after passage. When Caki and MSC cells were co-cultured together, there was the potential to form a sphere, and overlaps were frequently exhibited. These structures demonstrated that they could create a sphere in a three-dimensional environment and interact with each other cellularly. We found that when Caki and MSC cells are placed in alginate, they form a sphere in 3D culture medium when incubated for 21 days. When sections were taken from these spheres and the structures were examined, it was observed that clear cells exhibited organoid-like structures together with MSC cells, and morphologically changeable communities in kidney carcinoma cells were observed in HE staining in these structures (Figure 3).

**Figure 4:**
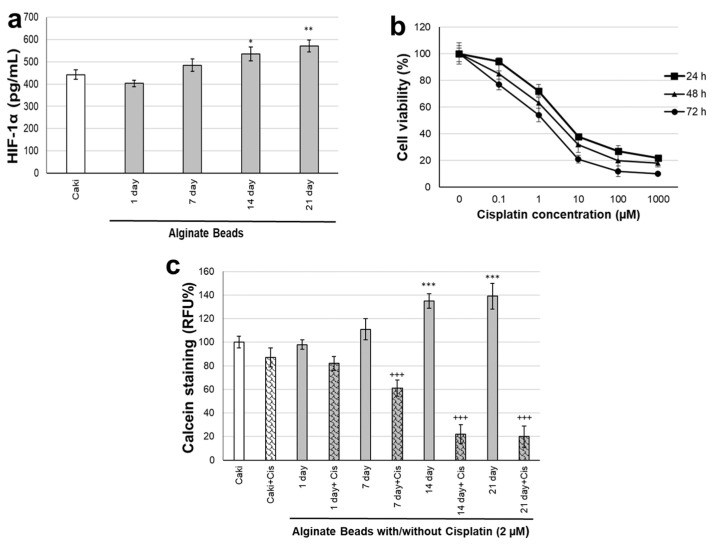
(**a**) HIF-1α protein levels of renal organoid cultured from day 1 to day 21 (* *p* ˂ 0.05, ** *p* ˂ 0.01 compared to Caki control cells). (**b**) Cell viability of cisplatin treatment with Caki cells at 24 h, 48 h, and 72 h. (**c**) Calcein staining of alginate renal organoid cultured from day 1 to day 21 (*** *p* ˂ 0.001 compared to Caki control cells, ^+++^ *p* ˂ 0.001 compare to alginate organoid without cisplatin for each day).

HIF-1α is a highly expressed marker in renal cancer cells. Therefore, it was checked whether the HIF-1α expression of cancer cells would continue even when encapsulated in alginate. When organoid modeling was performed in Caki and MSC cells in alginate, HIF-1α protein levels were measured on the 1st, 7th, 14th, and 21st days. HIF-1α levels did not change in renal organoids on day 1, increased on day 7 but were not significant, and increased significantly on days 14 and 21 (Figure 4a).

Cisplatin is a chemotherapeutic used as standard therapy in renal cancer. Therefore, cisplatin was used to measure drug response in organoid modeling with Caki and MSC cells in alginate. First, the IC50 value of cisplatin at 24 h, 48 h, and 72 h, depending on time, was calculated on Caki cells (Figure 4b). The IC50 concentration of cisplatin in Caki cells was found as 7.24 ± 2.16 µM, 5.23 ± 2.44 µM, and 2.21 ± 0.76 µM at 24 h, 48 h, and 72 h, respectively.

Since the Cisplatin concentration would be used in long-term incubations, 2 µM was preferred, and Cisplatin was applied in the organoid model. Renal organoid modeling in alginate was determined by calcein staining of incubations with and without Cisplatin for 21 days (Figure 4c). In calcein staining, cells were removed from alginate with a solubilization buffer, and viable cell numbers were controlled by determining the fluorescent esterase activity of living cells. The viability of the cells in alginate started to increase by about 40% after the 7th day, which was found to be significant (*p* ˂ 0.001). Cell viability decreased significantly in the cisplatin-treated organoids compared to the organoids without Cisplatin. In the cisplatin-administered groups, organoids decreased on the 1st day but were not found to be significant and decreased significantly on the 7th, 14th, and 21st days (*p* ˂ 0.001).

### 3.3. CXCR4 and CXCL-12 Changes on Renal Organoids

**Figure 5:**
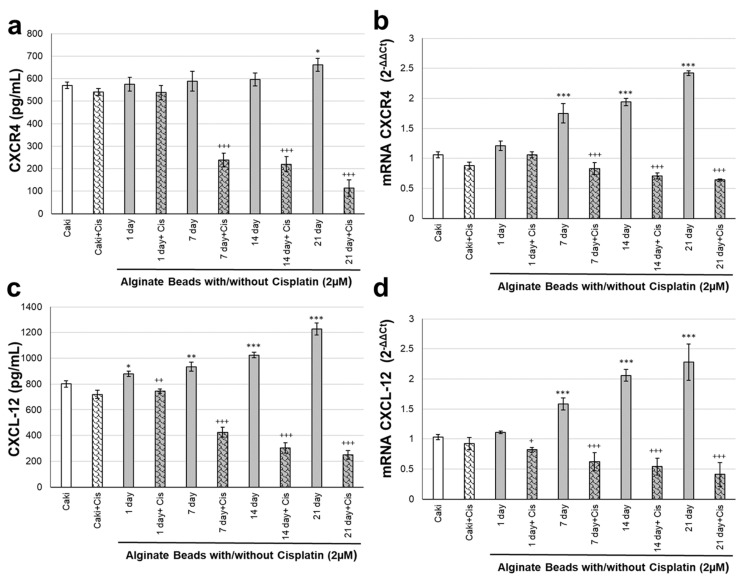
Tumor microenvironment response with/without Cisplatin in renal organoid modeling in alginate. (**a**) CXCR4 protein levels; (**b**) CXCR4 gene expression levels; (**c**) CXCL-12 protein expression levels; (**d**) CXCL-12 gene expression levels with/without Cisplatin in renal organoid modeling on the 1st, 7th, 14th, and 21st days (* *p* ˂ 0.05, ** *p* ˂ 0.01, *** *p* ˂ 0.001 compared to Caki control cells, ^+^ *p* ˂ 0.05, ^++^ *p* ˂ 0.01, and ^+++^ *p* ˂ 0.001 compared to alginate organoid without cisplatin for each day).

The levels of chemokine family members CXCR4 and CXCL-12, essential markers in the cancer microenvironment, were evaluated for protein and gene expressions. CXCR4 and CXCL-12 responses to cisplatin were monitored for 21 days in renal cancer organoid modeling in alginate (Figure 5). CXCR4 protein and gene levels did not change much in the Caki + C group incubated with cisplatin for 24 h compared to the Caki group. The CXCR4 protein levels of organoids made in alginate beads groups increased significantly only on the 21st day (*p* ˂ 0.05). In alginate beads organoid groups, cisplatin administration significantly decreased CXCR4 protein levels after the 7th, 14th and 21st day (*p* ˂ 0.001, Figure 5a). CXCR4 gene expression levels increased in organoids in alginate beads on days 7, 14, and 21 (*p* ˂ 0.001). Cisplatin application to alginate beads organoids significantly decreased CXCR4 gene expression levels on days 7, 14, and 21 (*p* ˂ 0.001, Figure 5b). CXCL-12 protein and gene levels did not change much in the Caki + Cis group incubated with cisplatin for 24 h compared to the Caki group. CXCL-12 protein expression levels increased significantly on the 1st, 7th, 14th, and 21st days of organoids made in alginate beads groups (*p* ˂ 0.05, *p* ˂ 0.01, *p* ˂ 0.001). In alginate beads organoid groups, cisplatin administration significantly decreased CXCL-12 protein expression levels on day 1, 7, 14, and 21 (*p* ˂ 0.001, Figure 5c). CXCL-12 gene expression levels increased in organoids in alginate beads on days 7, 14, and 21 (*p* ˂ 0.001). Cisplatin application to alginate beads organoids significantly decreased CXCR4 gene expression levels on the 1st, 7th, 14th, and 21st days (*p* ˂ 0.05, *p* ˂ 0.001, Figure 5d).

## 4. Discussion

Microfluidic systems have been rapidly integrated into organoid studies especially in cancer research [35]. This swift adoption of the lab-on-chip approach in organoids formation is stemming from highly precise sample manipulation capabilities of microfluidic devices. For example, Schuster et al. developed an automated microfluidic platform to study the drug response of human-derived pancreatic tumor organoids, which showed the potential of the on-chip drug screening in enabling personalized treatment strategies [36]. While their microfluidic platform enabled controlled on-chip organoid research, fabrication of the main microfluidic channel relied on expensive and time-consuming microfabrication procedures. In comparison, we used a much faster approach to fabricate the microfluidic device that was used to generate uniform-size cell-laden hydrogels for tumor organoid drug testing. Therefore, it is shown that the application of laser-prototyping is rapid and low-cost, which makes this fabrication method accessible for low-resourced environments. Studies on microfluidic systems and organoids in the literature are mostly aimed at increasing the viability of cells in the fluid system for measuring drug responses over a longer period of time. In our study, we wanted to emphasize that the reproducible experimental sets could be established with the uniformly sized organoids using a simple microfluidic system in drug testing studies. 

Chemokines are a group member of the g protein-coupled receptor family and are expressed in many cells. CXCR4 is an important chemokine that is overexpressed in cancer cells and is involved in the proliferation of cancer cells [37]. It has even been reported that CXCR4 levels are increased even in differentiated stem cells [38]. CXCL-12, also known as SDF-1, the ligand of CXCR4, has a very high affinity for glycosamines on the cell surface [39]. CXCL-12 is a specific filament of CXCR4, and although it binds, it is known to bind to different receptors, affect the signals of chemokines, and cause cell migration [40]. It is thought that CXCR4 and CXCL-12 can be evaluated together in terms of the tumor microenvironment and used in microenvironment modeling in drug studies. It is reported that the expression of CXCR4/CXCL12 is increased due to the hypoxic tumor environment, which is HIF-lα dependent [41]. As the amount of CXCL12 expression increases, the amount of CXCR4 increases in endothelial cancer cells in the tumor microenvironment and affects the cells forming spheroids [42]. CXCR4 is expressed in cancer stem cell populations in tumors, as it contributes to developing drug resistance and confers invasive and metastatic properties in renal cancer cells [43]. It is demonstrated that CXCL12/CXCR4 is essential in the formation of the sphere, where endothelial and stem cell proliferation occurs collaboratively. Our study used CXCR4 and CXCL-12 to model the tumor microenvironment versus drug response. Determining whether drugs are active in the tumor with 2D cultures and modeling the microenvironment bring some limitations. The use of organoid models is beneficial as a more extended examination of the effects of drugs will reveal the physiological situation in cancer treatments. We showed that when cisplatin is used in standard therapy renal organoid modeling, monitoring its effects at 21 days is vital for tumor cells and microenvironment evaluation. Analyzing the change in CXCR4 and CXCL-12 protein and gene levels over 21 days indicated that our renal organoid modeling might be more suitable for drug trial studies.

## Data Availability

The data is available at reasonable request from the corresponding authors.

## References

[1] Drost J., Clevers H. (2017). Translational applications of adult stem cell-derived organoids. Development.

[2] Sato T., Stange D.E., Ferrante M., Vries R.G.J., Van Es J.H., Van Den Brink S., Van Houdt W.J., Pronk A., Van Gorp J., Siersema P.D. (2011). Long-term expansion of epithelial organoids from human colon, adenoma, adenocarcinoma, and Barrett’s epithelium. Gastroenterology.

[3] Drost J., Clevers H. (2018). Organoids in cancer research. Nat. Rev. Cancer.

[4] Sachs N., Clevers H. (2014). Organoid cultures for the analysis of cancer phenotypes. Curr. Opin. Genet. Dev..

[5] Fendler A., Bauer D., Busch J., Jung K., Wulf-Goldenberg A., Kunz S., Song K., Myszczyszyn A., Elezkurtaj S., Erguen B. (2020). Inhibiting WNT and NOTCH in renal cancer stem cells and the implications for human patients. Nat. Commun..

[6] Calandrini C., Schutgens F., Oka R., Margaritis T., Candelli T., Mathijsen L., Ammerlaan C., van Ineveld R.L., Derakhshan S., de Haan S. (2020). An organoid biobank for childhood kidney cancers that captures disease and tissue heterogeneity. Nat. Commun..

[7] Eggener S.E., Yossepowitch O., Pettus J.A., Snyder M.E., Motzer R.J., Russo P. (2006). Renal Cell Carcinoma Recurrence After Nephrectomy for Localized Disease: Predicting Survival From Time of Recurrence. J. Clin. Oncol..

[8] Jonasch E., Gao J., Rathmell W.K. (2014). Renal cell carcinoma. BMJ.

[9] Hoefflin R., Harlander S., Schäfer S., Metzger P., Kuo F., Schönenberger D., Adlesic M., Peighambari A., Seidel P., Chen C. (2020). HIF-1α and HIF-2α differently regulate tumour development and inflammation of clear cell renal cell carcinoma in mice. Nat. Commun..

[10] Liu M., Cardilla A., Ngeow J., Gong X., Xia Y. (2022). Studying Kidney Diseases Using Organoid Models. Front. Cell Dev. Biol..

[11] Wood L. (2012). Sunitinib malate for the treatment of renal cell carcinoma. Expert Opin. Pharmacother..

[12] Grassi L., Alfonsi R., Francescangeli F., Signore M., De Angelis M.L., Addario A., Costantini M., Flex E., Ciolfi A., Pizzi S. (2019). Organoids as a new model for improving regenerative medicine and cancer personalized therapy in renal diseases. Cell Death Dis..

[13] Kim D., Dressler G.R. (2005). Nephrogenic Factors Promote Differentiation of Mouse Embryonic Stem Cells into Renal Epithelia. J. Am. Soc. Nephrol..

[14] Taguchi A., Nishinakamura R. (2017). Higher-Order Kidney Organogenesis from Pluripotent Stem Cells. Cell Stem Cell.

[15] Yoshida G.J. (2020). Applications of patient-derived tumor xenograft models and tumor organoids. J. Hematol. Oncol..

[16] Bolck H.A., Corrò C., Kahraman A., von Teichman A., Toussaint N.C., Kuipers J., Chiovaro F., Koelzer V.H., Pauli C., Moritz W. (2021). Tracing Clonal Dynamics Reveals that Two- and Three-dimensional Patient-derived Cell Models Capture Tumor Heterogeneity of Clear Cell Renal Cell Carcinoma. Eur. Urol. Focus.

[17] Li Z., Xu H., Yu L., Wang J., Meng Q., Mei H., Cai Z., Chen W., Huang W. (2022). Patient-derived renal cell carcinoma organoids for personalized cancer therapy. Clin. Transl. Med..

[18] Guo F., Li P., French J.B., Mao Z., Zhao H., Li S., Nama N., Fick J.R., Benkovic S.J., Huang T.J. (2015). Controlling cell–cell interactions using surface acoustic waves. Proc. Natl. Acad. Sci. USA.

[19] Wu Y., Ao Z., Chen B., Muhsen M., Bondesson M., Lu X., Guo F. (2018). Acoustic assembly of cell spheroids in disposable capillaries. Nanotechnology.

[20] Regmi S., Poudel C., Adhikari R., Luo K.Q. (2022). Applications of Microfluidics and Organ-on-a-Chip in Cancer Research. Biosensors.

[21] Homan K.A., Gupta N., Kroll K.T., Kolesky D.B., Skylar-Scott M., Miyoshi T., Mau D., Valerius M.T., Ferrante T., Bonventre J.V. (2019). Flow-enhanced vascularization and maturation of kidney organoids in vitro. Nat. Methods.

[22] Man Y., Maji D., An R., Ahuja S.P., Little J.A., Mohseni P., Suster M.A., Gurkan U.A. (2020). Assessment of Red Blood Cell-Mediated Microvascular Occlusion in Sickle Cell Disease By a Novel Electrical Impedance-Based Microfluidic Device. Blood.

[23] Man Y., An R., Monchamp K., Sekyonda Z., Kucukal E., Federici C., Wulftange W.J., Goreke U., Bode A., Sheehan V.A. (2022). OcclusionChip: A functional microcapillary occlusion assay complementary to ektacytometry for detection of small-fraction red blood cells with abnormal deformability. Front. Physiol..

[24] Wulftange W.J., Kucukal E., Man Y., An R., Monchamp K., Sevrain C.D., Dashora H.R., Owusu-Ansah A.T., Bode A., Ilich A. (2022). Antithrombin-III mitigates thrombin-mediated endothelial cell contraction and sickle red blood cell adhesion in microscale flow. Br. J. Haematol..

[25] Man Y., Kucukal E., An R., Bode A., Little J.A., Gurkan U.A. (2021). Standardized microfluidic assessment of red blood cell–mediated microcapillary occlusion: Association with clinical phenotype and hydroxyurea responsiveness in sickle cell disease. Microcirculation.

[26] Borók A., Laboda K., Bonyár A. (2021). PDMS Bonding Technologies for Microfluidic Applications: A Review. Biosensors.

[27] Ao Z., Cai H., Wu Z., Song S., Karahan H., Kim B., Lu H.-C., Kim J., Mackie K., Guo F. (2021). Tubular human brain organoids to model microglia-mediated neuroinflammation. Lab Chip.

[28] Cai H., Ao Z., Wu Z., Song S., Mackie K., Guo F. (2021). Intelligent acoustofluidics enabled mini-bioreactors for human brain organoids. Lab Chip.

[29] Ao Z., Cai H., Wu Z., Ott J., Wang H., Mackie K., Guo F. (2021). Controllable fusion of human brain organoids using acoustofluidics. Lab Chip.

[30] Fang G., Lu H., Rodriguez de la Fuente L., Law A.M.K., Lin G., Jin D., Gallego-Ortega D. (2021). Mammary Tumor Organoid Culture in Non-Adhesive Alginate for Luminal Mechanics and High-Throughput Drug Screening. Adv. Sci..

[31] Abas B.I., Demirbolat G.M., Cevik O. (2022). Wharton jelly-derived mesenchymal stem cell exosomes induce apoptosis and suppress EMT signaling in cervical cancer cells as an effective drug carrier system of paclitaxel. PLoS ONE.

[32] Abas B.I., Cevik E., Kocabiyik B., Cenik M., Cevik O., Gumus E. (2021). Alginate encapsulation induce colony formation with umbilical cord-derived mesenchymal stem cells. Exp. Biomed. Res..

[33] Akkoyun F., Ozcelik A. (2020). A Simple Approach for Controlling an Open-Source Syringe Pump. Eur. Mech. Sci..

[34] Galateanu B., Dimonie D., Vasile E., Nae S., Cimpean A., Costache M. (2012). Layer-shaped alginate hydrogels enhance the biological performance of human adipose-derived stem cells. BMC Biotechnol..

[35] Duzagac F., Saorin G., Memeo L., Canzonieri V., Rizzolio F. (2021). Microfluidic Organoids-on-a-Chip: Quantum Leap in Cancer Research. Cancers.

[36] Schuster B., Junkin M., Kashaf S.S., Romero-Calvo I., Kirby K., Matthews J., Weber C.R., Rzhetsky A., White K.P., Tay S. (2020). Automated microfluidic platform for dynamic and combinatorial drug screening of tumor organoids. Nat. Commun..

[37] Zlotnik A. (2006). Chemokines and cancer. Int. J. Cancer.

[38] Chen Y., Wei Y., Liu J., Zhang H. (2014). Chemotactic Responses of Neural Stem Cells to SDF-1α Correlate Closely with Their Differentiation Status. J. Mol. Neurosci..

[39] Righetti A., Giulietti M., Šabanović B., Occhipinti G., Principato G., Piva F. (2019). CXCL12 and Its Isoforms: Different Roles in Pancreatic Cancer?. J. Oncol..

[40] Tirone M., Tran N.L., Ceriotti C., Gorzanelli A., Canepari M., Bottinelli R., Raucci A., Di Maggio S., Santiago C., Mellado M. (2018). High mobility group box 1 orchestrates tissue regeneration via CXCR4. J. Exp. Med..

[41] Zhu C., Yao W.-L., Tan W., Zhang C.-H. (2017). SDF-1 and CXCR4 play an important role in adult SVZ lineage cell proliferation and differentiation. Brain Res..

[42] Kryczek I., Lange A., Mottram P., Alvarez X., Cheng P., Hogan M., Moons L., Wei S., Zou L., Machelon V. (2005). CXCL12 and vascular endothelial growth factor synergistically induce neoangiogenesis in human ovarian cancers. Cancer Res..

[43] Gassenmaier M., Chen D., Buchner A., Henkel L., Schiemann M., Mack B., Schendel D.J., Zimmermann W., Pohla H. (2013). CXC Chemokine Receptor 4 is Essential for Maintenance of Renal cell Carcinoma-Initiating Cells and Predicts Metastasis. Stem Cells.

